# Association of prior criminal charges and convictions with subsequent violent and firearm-related crime: a retrospective cohort study

**DOI:** 10.1186/s40621-025-00593-x

**Published:** 2025-07-01

**Authors:** Julia P. Schleimer, Rachel Ross, Ali Rowhani-Rahbar

**Affiliations:** 1 Department of Epidemiology, School of Public Health, University of Washington, Seattle, WA USA; 2 Firearm Injury & Policy Research Program, School of Medicine, University of Washington, Seattle, WA USA

**Keywords:** Firearms, Violence, Domestic violence, Criminal justice

## Abstract

**Background:**

Interpersonal violence is a pressing public health problem in the United States. Those who interact with the criminal legal system are likely to experience multiple intersecting risks that increase the probability of subsequent violence perpetration. To inform development and implementation of risk-reduction interventions within the criminal legal system or for those involved in it, there is a need for contemporary population-level estimates of violence perpetration risk among those with prior criminal charges or convictions.

**Methods:**

In this state-wide retrospective cohort study of individuals in Washington state, we estimated the risk of future violent and firearm-related charges and convictions among those with misdemeanor charges or convictions for violence-related (including domestic violence), firearm-related, and substance use-related offenses from 2015 to 2019 compared to those with infractions. Subdistribution Hazard Ratios (sdHR) were used to quantify relative risk.

**Results:**

Sample sized varied across comparisons, ranging from 766 people with an index firearm-related conviction to 1,280,070 with an index infraction. Relative risk of outcomes ranged from 11.73 (95% CI = 9.08–15.14) (comparing risk of subsequent violent crime conviction among those with index drug/alcohol misdemeanor convictions vs. infractions) to 155.23 (95% CI = 136.87–176.07) (comparing risk of subsequent violent crime conviction among those with index domestic violence-related misdemeanor convictions vs. infractions). Individuals with domestic violence-related misdemeanor convictions had over 30 times the risk of subsequent firearm-related charges and convictions compared to those with infractions.

**Conclusions:**

Findings from this state-wide study inform opportunities to reduce risk for subsequent violence and firearm-related harm, for example through tailored intervention, investment in healing-centered deflection strategies, and improved implementation of domestic violence firearm prohibitions.

**Supplementary Information:**

The online version contains supplementary material available at 10.1186/s40621-025-00593-x.

## Background

Interpersonal violence (including abuse, intimate partner violence, sexual violence, and community violence) is a leading contributor to morbidity and mortality in the United States (US), with profound physical, psychosocial, and economic consequences for individuals and communities [1, 2]. Interpersonal firearm violence in particular has an outsized burden and severe consequences [3–5]. In 2023, 17,927 of 22,829 homicides in the US involved firearms (79%), and 54% of US adults reported either having been a victim of firearm violence themselves or having a family member impacted by a firearm-related incident [6, 7].

Identifying risk factors for interpersonal (firearm) violence perpetration can help inform the development and implementation of interventions to reduce risk. Prior research suggests those who interact with the criminal legal system are especially likely to experience multiple intersecting risks (e.g., prior violence exposure and perpetration, substance use), often shaped by social and structural factors, that are associated with increased probability of subsequent violence perpetration [8, 9]. Indeed, decades of research in criminology has found that prior criminal history is an important predictor of future violent arrest, charge, and conviction [10–16]. For example, in a 1996 meta-analysis of 131 studies, Gendreau et al. found that prior criminal history was one of the strongest correlates of recidivism across a range of sociodemographic, family, and behavioral indicators [17]. In a 2012 review of the literature on violent crime throughout the lifecourse, Piquero et al. found that a small group of people with chronic criminal legal system involvement (of many offense types) are responsible for most violent offenses [18]. Further, in a study of trauma patients in Washington state from 2001 to 2011, individuals with a history of arrest for a firearm-related or violent crime had almost 4 times the risk of subsequent firearm- or violence-related arrest compared to those without such prior criminal history [19]. National estimates suggest that between 58% and 63% of incarcerated individuals met criteria for a substance use disorder, and 37–42% reported being intoxicated at the time of the offense for which they were arrested [20]. 

However, contemporary population-level estimates of violence perpetration risk among those with prior criminal charges or convictions are lacking in several states, including Washington state, the focus of the current study. Such estimates could help identify gaps in need, inform state and local policies and programs, and ground future effectiveness evaluations of interventions within the criminal legal system or for those involved in it. For example, studies of individuals who legally purchased a handgun in California in 2001 found that those with vs. without prior convictions for alcohol-related offenses had approximately 2–3 times the risk of subsequent arrest for violent or firearm-related offenses, suggesting opportunities for tailored risk reduction strategies [21, 22]. Other research suggests the promise of interventions that deflect individuals away from the criminal legal system towards community-based services and facilitate connection to needed care, including substance use disorder treatment; identifying populations within the criminal legal system at particularly elevated risk for subsequent violence could guide implementation of such interventions [23, 24]. Further, several existing firearm prohibitions are based on criminal history (e.g., people with domestic violence misdemeanor convictions are prohibited from purchasing and possessing firearms, 18 U.S.C. § 922(g)(9)), so examination of risk of firearm-related and violent crime among individuals meeting prohibiting criteria could shed light on how those policies are being implemented.

In this state-wide retrospective cohort study of individuals in Washington state, we estimated the risk of future violent and firearm-related charges and convictions among those with misdemeanor charges or convictions for violent (including domestic violence), firearm-related, and substance use-related offenses from 2015 to 2019 compared to those with infractions. Results will inform opportunities to better support individuals involved in the criminal legal system and promote public safety.

## Methods

### Study design, population, and setting

This was a retrospective cohort study of individuals aged 18 and older with an infraction (in Washington municipal or district courts) or a criminal charge or conviction (in Washington district or superior courts) for violent, firearm-related, or substance use-related misdemeanors in Washington state from 2015 to 2019, with follow up through 2020.

### Exposures

We examined eight exposures: misdemeanor charges for violent, domestic violence-related (a subset of all violent offenses), firearm-related, and drug/alcohol-related offenses; and misdemeanor convictions for violent, domestic violence-related, firearm-related, and drug/alcohol-related offenses. Data were obtained from the Washington State Administrative Office of the Courts, and offenses were categorized per the Revised Code of Washington (RCW), municipal codes, and information about domestic violence contained in structured fields. Each exposure was compared to infractions, which included traffic (e.g., speeding) and non-traffic (e.g., littering) related violations and which we used to approximate risk among the general population. To construct these comparisons, we separately selected each person’s first (“index”) infraction or misdemeanor for each offense/exposure type (thus allowing individuals to contribute observations to each misdemeanor type). As such, the sample size for the infraction group varied across comparisons.

As done in prior research [19, 25], we defined violent crime in two ways, using: (1) the Federal Bureau of Investigation Uniform Crime Reporting (UCR) program definition (“UCR violent crime”), and, (2) a more expansive conceptualization of violence (“any violent crime”) [26]. We identified offenses as firearm related if they included any firearm-related RCW or municipal code. With minor exception, such codes only identify non-violent crimes such as theft of a firearm and violations related to firearm possession, carrying, and sales. Drug/alcohol-related offenses were defined as any offense involving the actual or attempted use, possession, or sale/distribution of alcohol, any drug (including cigarettes/tobacco), or related paraphernalia. We excluded 4.6% of misdemeanor observations due to missing name (required for linkage, described below) and 9.5% of misdemeanor observations due to missing information on penalty type (e.g., misdemeanor or felony), required to define exposure.

### Outcomes

Outcomes were new violent and firearm-related charges and convictions (either misdemeanor or felony) following the index offense through 2020 in Washington district or superior courts. Violent and firearm-related crime were defined as above.

### Analysis

We descriptively characterized the study population (using administratively collected demographic data) and estimated the cumulative incidence of outcomes per exposure group, treating death and incarceration prior to outcomes of interest as competing events, otherwise censoring individuals at the end of the study period (12/31/2020). We ascertained death dates from Washington Department of Health (data were missing for the fourth quarter of 2020 because the Department of Health stopped processing requests due to the COVID-19 pandemic). We determined incarceration with adult felony sentencing data provided by the Washington State Caseload Forecast Council and, as in prior research [25], considered individuals to be incarcerated from sentence date onward. We additionally used the Fine-Gray method to estimate Subdistribution Hazard Ratios (sdHR) for outcomes, comparing those with misdemeanors to those with infractions. Because this was a descriptive study, we did not include covariates in regression models.

Data were probabilistically linked with Link King (a SAS/AF application for record linkage and unduplication) in SAS version 9.4 (SAS Institute, Cary NC) using individual name and date of birth, including information on gender and race and ethnicity when available.

Analyses were done in R version 4.0.0 (R Foundation for Statistical Computing, Vienna, Austria), using the “cmprsk” package (version 2.2–11). The University of Washington Institutional Review Board approved this study and waived informed consent.

## Results

### Description of study population

There were 63,228 people with an index misdemeanor charge for a violent offense, 46,215 for a domestic violence-related offense, 2,232 for a firearm-related offense, and 88,897 for a drug/alcohol-related offense (Table 1). There were 29,086 people with an index misdemeanor conviction for a violent offense, 19,487 for a domestic violence-related offense, 766 for a firearm-related offense, and 45,282 for a drug/alcohol-related offense (Additional File 1 eTable 1). The number of people with index infractions ranged from 1,274,940 to 1,280,070, depending on the misdemeanor comparison. Those with index misdemeanor charges and convictions were generally more likely to be identified as male, American Indian or Alaskan Native, and Black than those with infractions (Table 1, Additional File 1 eTable 1).

**Table 1:** Description of individuals with index misdemeanor charges or infractions in Washington state, 2015–2019

	Any violent misdemeanor charge (N = 63228)	Infraction (N = 1277596)^b^
*Age at case file (years)*		
Missing	3	2766
Mean (SD)	35.69 (12.47)	38.38 (15.10)
Range	18.00–98.00	18.00–99.53
*Sex*		
Female	14,989 (23.7%)	467,761 (36.6%)
Male	48,088 (76.1%)	808,032 (63.2%)
Unknown	151 (0.2%)	1803 (0.1%)
*Race* ^a^		
American Indian or Alaskan Native	1312 (2.1%)	11,043 (0.9%)
Asian	1622 (2.6%)	66,907 (5.2%)
Black	6270 (9.9%)	74,905 (5.9%)
Multiracial	110 (0.2%)	3349 (0.3%)
Native Hawaiian or Pacific Islander	25 (0.0%)	40 (0.0%)
Refused	0 (0.0%)	31 (0.0%)
Unknown	8079 (12.8%)	217,116 (17.0%)
White	45,799 (72.4%)	904,205 (70.8%)

	**Domestic violence-related misdemeanor charge** **(N = 46215)**	**Infraction** **(N = 1278227)** ^b^
*Age at case file (years)*		
Missing	2	2766
Mean (SD)	35.55 (12.06)	38.38 (15.10)
Range	18.00–98.00	18.00–99.53
*Sex*		
Female	11,221 (24.3%)	467,880 (36.6%)
Male	34,901 (75.5%)	808,543 (63.3%)
Unknown	93 (0.2%)	1804 (0.1%)
*Race* ^a^		
American Indian or Alaskan Native	874 (1.9%)	11,056 (0.9%)
Asian	1163 (2.5%)	66,915 (5.2%)
Black	4398 (9.5%)	74,943 (5.9%)
Multiracial	61 (0.1%)	3351 (0.3%)
Native Hawaiian or Pacific Islander	17 (0.0%)	40 (0.0%)
Refused	0 (0.0%)	31 (0.0%)
Unknown	5959 (12.9%)	217,207 (17.0%)
White	33,739 (73.0%)	904,684 (70.8%)

	**Firearm-related misdemeanor charge** **(N = 2232)**	**Infraction** **(N = 1279965)** ^b^
*Age at case file (years)*		
Missing	0	2766
Mean (SD)	35.68 (14.41)	38.38 (15.09)
Range	18.00–88.00	18.00–99.53
*Sex*		
Female	171 (7.7%)	468,314 (36.6%)
Male	2055 (92.1%)	809,844 (63.3%)
Unknown	6 (0.3%)	1807 (0.1%)
*Race* ^a^		
American Indian or Alaskan Native	53 (2.4%)	11,072 (0.9%)
Asian	64 (2.9%)	66,947 (5.2%)
Black	198 (8.9%)	75,057 (5.9%)
Multiracial	6 (0.3%)	3356 (0.3%)
Native Hawaiian or Pacific Islander	1 (0.0%)	40 (0.0%)
Refused	0 (0.0%)	31 (0.0%)
Unknown	279 (12.5%)	217,432 (17.0%)
White	1631 (73.1%)	906,030 (70.8%)

	**Drug/alcohol-related misdemeanor charge** **(N = 88897)**	**Infraction** **(N = 1274940)** ^b^
*Age at case file (years)*		
Missing	4	2766
Mean (SD)	34.41 (13.27)	38.39 (15.10)
Range	18.00–93.00	18.00–99.53
*Sex*		
Female	24,366 (27.4%)	467,072 (36.6%)
Male	64,445 (72.5%)	806,065 (63.2%)
Unknown	86 (0.1%)	1803 (0.1%)
*Race* ^a^		
American Indian or Alaskan Native	1986 (2.2%)	10,986 (0.9%)
Asian	2416 (2.7%)	66,835 (5.2%)
Black	5599 (6.3%)	74,818 (5.9%)
Multiracial	243 (0.3%)	3330 (0.3%)
Native Hawaiian or Pacific Islander	9 (0.0%)	40 (0.0%)
Refused	1 (0.0%)	31 (0.0%)
Unknown	13,752 (15.5%)	216,555 (17.0%)
White	64,891 (73.0%)	902,345 (70.8%)

^a^Ethnicity data were missing for most people and so are not presented

^b^The sample size for the infraction group varied across comparisons because we separately selected each person’s first (“index”) infraction or misdemeanor for each offense/exposure type (thus allowing individuals to contribute observations to each misdemeanor type)

### Prior misdemeanor charges and subsequent violent and firearm-related charges and convictions

**Fig. 1:**
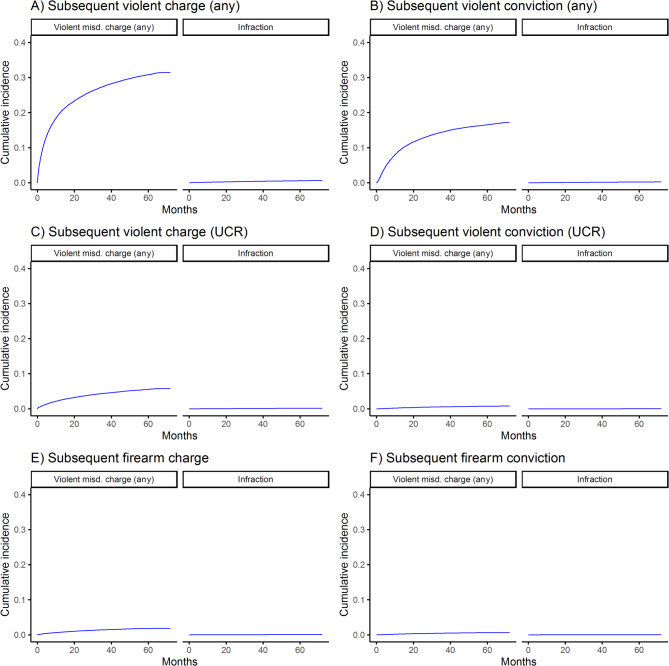
Incidence of subsequent violent and firearm-related charges and convictions among those with prior violent misdemeanor charges (any violent crime) or infractions, misd. = misdemeanor; UCR = uniform crime reporting

**Fig. 2:**
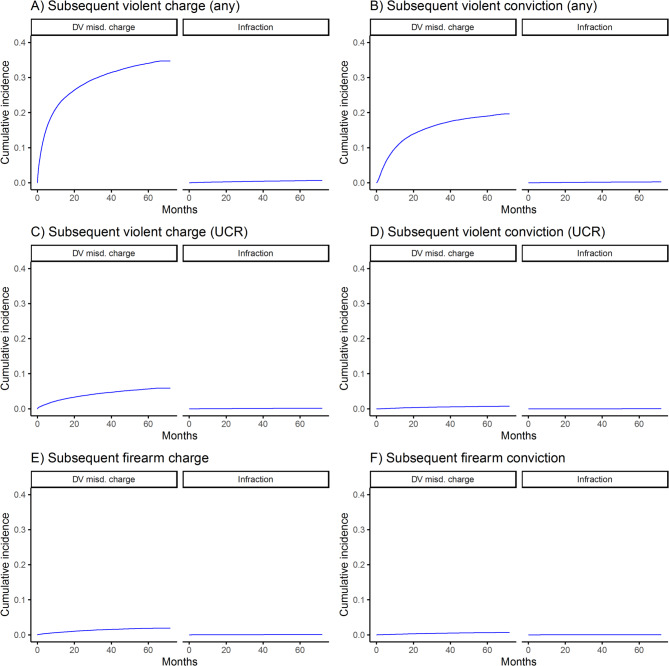
Incidence of subsequent violent and firearm-related charges and convictions among those with prior domestic violence misdemeanor charges or infractions, DV = domestic violence; misd. = misdemeanor; UCR = uniform crime reporting

**Fig. 3:**
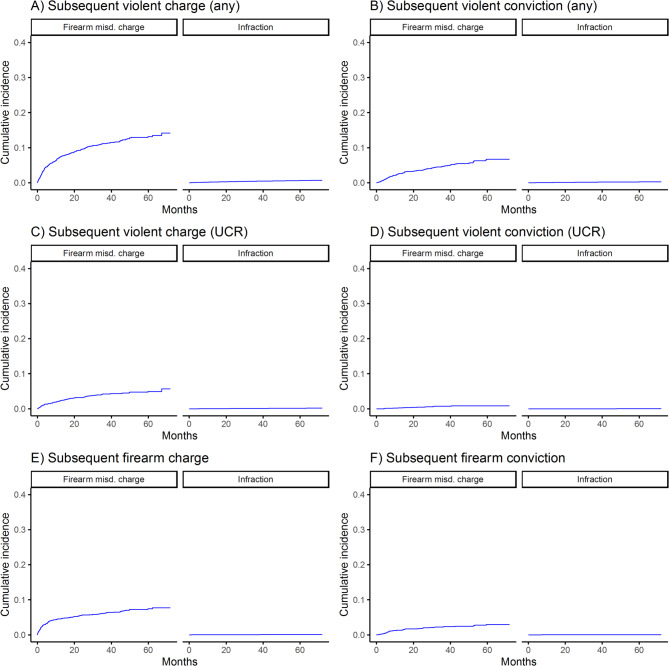
Incidence of subsequent violent and firearm-related charges and convictions among those with prior firearm-related misdemeanor charges or infractions, misd. = misdemeanor; UCR = uniform crime reporting

**Fig. 4:**
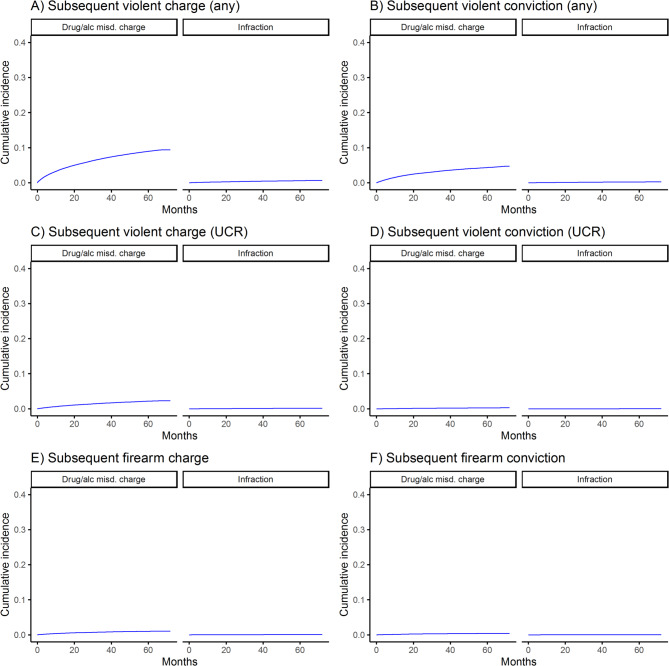
Incidence of subsequent violent and firearm-related charges and convictions among those with prior drug/alcohol-related misdemeanor charges or infractions, alc = alcohol; misd. = misdemeanor; UCR = uniform crime reporting

The cumulative incidences of subsequent violent and firearm-related charges and convictions among those with prior misdemeanor charges or infractions are shown in Figs. 1, 2, 3 and 4. For all outcomes, cumulative incidence was higher among those with prior charges vs. infractions, and subsequent violent crime (any violent crime) was most common (e.g., by 5 years, 30.8% of those with prior violent misdemeanor charges had a subsequent such charge), followed by subsequent UCR violent crime and firearm-related crime. Counts and rates of outcomes per 1,000 person-years and sdHRs comparing the risk of outcomes among those with misdemeanor charges vs. infractions are shown in Table 2. Relative risk of outcomes ranged from 11.99 (95% CI = 9.74–14.77) (comparing risk of subsequent UCR violent crime conviction among those with index drug/alcohol misdemeanor charges vs. infractions) to 150.56 (95% CI = 80.2–282.66) (comparing risk of subsequent firearm-related crime conviction among those with index firearm-related misdemeanor charges vs. infractions), depending on the exposure and outcome.

**Table 2:** Association between prior misdemeanor charge and subsequent violent and firearm-related charges and convictions

Outcome	Count (rate)^a^		sdHR (95% CI)^b^
	**Any violent misdemeanor charge**	**Infraction**	**Any violent misdemeanor charge vs. infraction**
Subsequent violent charge (any violent crime)	17,688 (116.79)	5109 (1.28)	80.05 (71.67, 89.40)
Subsequent violent charge (UCR violent crime)	2934 (15.85)	1273 (0.32)	44.74 (38.43, 52.09)
Subsequent firearm-related charge	956 (5.10)	612 (0.15)	30.04 (27.11, 33.28)
Subsequent violent conviction (any violent crime)	9308 (54.82)	1714 (0.43)	114.53 (107.82, 121.67)
Subsequent violent conviction (UCR violent crime)	376 (2.00)	186 (0.05)	37.76 (31.44, 45.35)
Subsequent firearm-related conviction	320 (1.70)	187 (0.05)	32.26 (26.87, 38.74)
	**Domestic violence-related misdemeanor charge**	**Infraction**	**Domestic violence-related misdemeanor charge vs. infraction**
Subsequent violent charge (any violent crime)	14,420 (132.48)	5197 (1.31)	90.11 (87.02, 93.31)
Subsequent violent charge (UCR violent crime)	2198 (16.05)	1296 (0.33)	44.95 (40.50, 49.89)
Subsequent firearm-related charge	710 (5.11)	626 (0.16)	29.83 (26.76, 33.25)
Subsequent violent conviction (any violent crime)	7934 (64.45)	1814 (0.46)	128.42 (120.46, 136.91)
Subsequent violent conviction (UCR violent crime)	254 (1.82)	186 (0.05)	34.76 (11.43, 105.74)
Subsequent firearm-related conviction	233 (1.67)	192 (0.05)	31.07 (24.19, 39.90)
	**Firearm-related misdemeanor charge**	**Infraction**	**Firearm-related misdemeanor charge vs. infraction**
Subsequent violent charge (any violent crime)	254 (41.96)	5564 (1.40)	27.04 (23.81, 30.72)
Subsequent violent charge (UCR violent crime)	93 (14.61)	1361 (0.34)	37.81 (29.73, 48.08)
Subsequent firearm-related charge	144 (23.06)	645 (0.16)	129.53 (107.74, 155.74)
Subsequent violent conviction (any violent crime)	112 (17.78)	2086 (0.52)	30.21 (24.84, 36.75)
Subsequent violent conviction (UCR violent crime)	15 (2.33)	195 (0.05)	40.98 (22.58, 74.37)
Subsequent firearm-related conviction	52 (8.17)	189 (0.05)	150.56 (80.20, 282.66)
	**Drug/alcohol-related misdemeanor charge**	**Infraction**	**Drug/alcohol-related misdemeanor charge vs. infraction**
Subsequent violent charge (any violent crime)	6543 (23.35)	5304 (1.34)	17.07 (16.4, 17.76)
Subsequent violent charge (UCR violent crime)	1524 (5.25)	1301 (0.33)	15.49 (14.36, 16.71)
Subsequent firearm-related charge	749 (2.57)	628 (0.16)	16.15 (13.62, 19.15)
Subsequent violent conviction (any violent crime)	3191 (11.14)	1965 (0.49)	21.92 (20.71, 23.21)
Subsequent violent conviction (UCR violent crime)	177 (0.61)	189 (0.05)	11.99 (9.74, 14.77)
Subsequent firearm-related conviction	297 (1.02)	189 (0.05)	20.94 (17.39, 25.21)

^a^Rate per 1,000 person-years

^b^Estimated from unadjusted competing risk models

### Prior misdemeanor convictions and subsequent violent and firearm-related charges and convictions

The cumulative incidences of subsequent violent and firearm-related charges and convictions among those with prior misdemeanor convictions or infractions are shown in Additional File 1 eFigures 1–4. As for misdemeanor charges, the cumulative incidence for all outcomes was higher among those with prior misdemeanor convictions vs. infractions, and subsequent violent crime (any violent crime) and UCR violent crime were more common than firearm-related crime. sdHRs comparing the risk of outcomes among those with misdemeanor convictions vs. infractions (Additional File 1 eTable2) ranged from 11.73 (95% CI = 9.08–15.14) (comparing risk of subsequent UCR violent crime conviction among those with index drug/alcohol misdemeanor convictions vs. infraction) to 155.23 (95% CI = 136.87–176.07) (comparing risk of subsequent violent crime conviction among those with index domestic violence-related misdemeanor convictions vs. infractions), depending on the exposure and outcome. Those with firearm-related misdemeanor convictions had 131.64 times the risk of subsequent firearm-related conviction (95% CI = 79.18–218.88) compared to those with infractions.

## Discussion

This retrospective cohort study found that individuals with prior misdemeanor charges and convictions for violence-related (including domestic violence), firearm-related, and drug/alcohol-related offenses in Washington state had substantially higher risk of subsequent charges or convictions for violent or firearm-related crimes, including felonies, compared to those with minor infractions (e.g., speeding tickets). The absolute risk among those with prior misdemeanors was also high; for example, approximately 30% of those with a violent misdemeanor charge had a subsequent violent charge within 5 years. These estimates are similar to prior research (e.g., in a study of 34 states, 32% of those incarcerated for a violent offense were re-arrested for a violent offense within 5 years) [27]. Drug/alcohol-related misdemeanors were relatively weak (though still strong) indicators of subsequent violent and firearm-related crime, while violent misdemeanors (including domestic violence misdemeanors) were especially strong markers for subsequent violent crime, and firearm-related misdemeanors were especially strong markers for subsequent firearm-related crime, suggesting a need for more investment in prevention for these groups.

Our findings align with prior work suggesting that individuals with prior criminal history, including for violent and non-violent offenses such as substance use, are at elevated risk of subsequent violent and/or firearm-related crime perpetration. For example, in a review of the literature, Piquero found that those with violent offenses tended to have many arrests for other offenses [18]. In a series of studies of handgun purchasers in California, those with a history of charges or convictions for crimes such as simple and aggravated assault, weapons offenses, drug use offenses, and driving under the influence (DUI) were at heightened risk of subsequently being arrested for violent and firearm-related violent offenses compared to those without such criminal history [21, 28]. There are several possible reasons for the associations observed in these prior studies and in the current one, including that factors that contributed to initial/initially-measured criminal legal system involvement also contributed to subsequent involvement (e.g., risky behavior, economic precarity, heightened police surveillance) and/or that criminal legal system involvement itself acted as destabilizing force (e.g., harming employment and housing opportunities, mental health, and family and other relationships) thus contributing to subsequently elevated risk [29–31]. For example, in a review of quasi-experimental studies, Loeffler and Nagin found that incarceration, when not accompanied by rehabilitative programming, was associated with increased risk of re-arrest, charge, and conviction [32]. Indeed, research suggests criminal legal system involvement can elevate risk of subsequent offending by increasing exposure to criminally-involved peers, “labeling” individuals as socially-deviant (contributing to enacted and internalized stigma), and creating or exacerbating financial hardship [33–35]. Regardless of the causes for or mechanisms underlying the associations we observed, our findings suggest opportunities to better support individuals involved in the criminal legal system and reduce their risk of enacting future violence and firearm-related harm.

One strategy intended to reduce risk of firearm-related violence is limiting access to firearms. For example, individuals convicted of domestic violence misdemeanors are prohibited under federal law from purchasing or possessing firearms (18 U.S.C. § 922(g)(9)), and the US Supreme Court recently upheld the state’s right to temporarily restrict individuals’ access to firearms when they pose a credible threat to the safety of others [36]. Our results show that individuals with domestic violence misdemeanor convictions had 36 and 38 times the risk of subsequent firearm-related charges and convictions, respectively, compared to those with infractions. This suggests opportunities to improve implementation of the policy (notwithstanding the possibility that some firearm-related offenses may have been for violating the prohibition, which would suggest enforcement). Prior work on domestic violence firearm prohibitions suggests intentional implementation by dedicated practitioners is key to policy enforcement, compliance, and firearm relinquishment [37, 38]. Likewise, while federal law prohibits people who are “unlawful users of or addicted to a controlled substance,” it is well recognized that the law is poorly defined and difficult to implement [9]. Federal law does not restrict access to firearms on the basis of alcohol use (despite the strong link between alcohol use and firearm-related harm [39]), but Washington state law (implemented in 2023, after our study period) prohibits people with certain DUI offenses from purchasing or possessing firearms (Washington RCW 9.41.040(2)(a)(i)(D)). Our results provide context for understanding the implementation and potential effects of this law. Unlike some states, Washington state does not prohibit firearm purchase and possession for those with violent misdemeanor convictions, which prior research suggests meaningfully reduces risk [40]. 

In addition to firearm prohibition for people with certain criminal history (which requires enforcement and may thus perpetuate criminal legal system involvement), it is important to also consider more holistic, healing-centered strategies to reduce risk and meet people’s underlying needs. Such strategies could involve connection to social services, community-based organizations, and/or substance use treatment. For example, community violence intervention (CVI) is a non-punitive approach that seeks to reduce violence and support the healing, growth, and empowerment of individuals and groups at high risk of violence via a range of strategies, including mentorship and life coaching, cognitive behavioral intervention, wraparound supports and referrals, and connection to job training and employment opportunities [41]. The CVI workforce is primarily comprised of individuals with lived experiences of violence and incarceration; thus, the credible messenger model of CVI recognizes not only the relational power of lived experience but offers direct pathways to employment for people with prior criminal legal system involvement.

Specialty/problem-solving courts, deflection/diversion programs, and reentry interventions may also reduce risk and help support the needs of those involved in the criminal legal system [24, 42–44]. For example, research has found that Seattle’s Law Enforcement Assisted Diversion program, a deflection program for individuals with drug/alcohol-related offenses, was associated with improvements in housing, employment, and income/benefits for program participants [45]. A study of a reentry program in Minnesota designed to help individuals find stable employment upon release from incarceration found that the program was associated with increased likelihood of employment and higher wages [46]. 

### Limitations

Our data contained a small amount of missingness (e.g., on name and offense disposition). We focused on individuals’ index misdemeanor charge or conviction during the study period and did not have longitudinal data prior to the start of the study. We also lacked detail on individuals’ circumstances, including whether and what types of services they had access to, which could provide more specific information on gaps in service needs. Demographic information collected in the administrative datasets we used may be collected with error and not represent the full expression of individuals’ identities (e.g., our measures do not distinguish between self-identified race vs. other-perceived race). We treated death and incarceration as competing events in this study but lacked longitudinal data on state of residence over time; as such, cumulative incidence of outcomes may be underestimated. Finally, this study is specific to Washington state and, while local data are useful given differences in contexts and laws/policies across jurisdictions, our findings may not generalize.

## Conclusions

Results of this population-based retrospective cohort study in Washington state suggest that individuals with misdemeanor charges and convictions for violent (including domestic violence), firearm-related, and drug/alcohol-related offenses are at substantially elevated risk of subsequent violent and firearm-related crime compared to those with minor infractions. Findings inform opportunities to reduce risk for subsequent violent and firearm-related crime, for example through tailored intervention (such as for those with firearm-related misdemeanors, who had the highest relative risk of subsequent firearm-related crime), investment in healing-centered deflection strategies, and improved implementation of domestic violence firearm prohibitions.

## Electronic supplementary material

Addititional file 1: eTable 1. Description of individuals with index misdemeanor convictions or infractions in Washington State, 2015-2019; eTable 2. Association between prior misdemeanor conviction and subsequent violent and firearm-related charges and convictions; eFigure 1. Incidence of subsequent violent and firearm-related charges and convictions among those with prior violent misdemeanor convictions or infractions; eFigure 2. Incidence of subsequent violent and firearm-related charges and convictions among those with prior domestic violence misdemeanor convictions or infractions; eFigure 3. Incidence of subsequent violent and firearm-related charges and convictions among those with prior firearm-related misdemeanor convictions or infractions; eFigure 4. Incidence of subsequent violent and firearm-related charges and convictions among those with prior drug/alcohol-related misdemeanor convictions or infractions

## Data Availability

The data that support the findings of this study are available from the Washington Administrative Offices of the Courts but restrictions apply to the availability of these data, which were used under license for the current study, and so are not publicly available.

## Abbreviations

sdHR: Subdistribution Hazard Ratio
CVI: Community violence intervention
DUI: Driving under the influence
RCW: Revised Code of Washington
UCR: Uniform Crime Reporting
