# Supplementary material for: Association of prior criminal charges and convictions with subsequent violent and firearm-related crime: a retrospective cohort study
Source: Inj Epidemiol. 2025 Jul 1;12:35. doi: 10.1186/s40621-025-00593-x (PMC12219223; doi:10.1186/s40621-025-00593-x)
Supplement: Supplementary file 1 — Addititional file 1: eTable 1. Description of individuals with index misdemeanor convictions or infractions in Washington State, 2015-2019; eTable 2. Association between prior misdemeanor conviction and subsequent violent and firearm-related charges and convictions; eFigure 1. Incidence of subsequent violent and firearm-related charges and convictions among those with prior violent misdemeanor convictions or infractions; eFigure 2. Incidence of subsequent violent and firearm-related charges and convictions among those with prior domestic violence misdemeanor convictions or infractions; eFigure 3. Incidence of subsequent violent and firearm-related charges and convictions among those with prior firearm-related misdemeanor convictions or infractions; eFigure 4. Incidence of subsequent violent and firearm-related charges and convictions among those with prior drug/alcohol-related misdemeanor convictions or infractions [file 40621_2025_593_MOESM1_ESM.docx]

**Additional File 1**

Association of prior criminal charges and convictions with subsequent violent and firearm-related crime: A retrospective cohort study

Julia P. Schleimer,^a,b^ Rachel Ross,^b^ Ali Rowhani-Rahbar^a,b^

^a^Department of Epidemiology, School of Public Health, University of Washington, Seattle, WA, USA

^b^Firearm Injury & Policy Research Program, School of Medicine, University of Washington, Seattle, WA, USA

eTable 1.Description of individuals with index misdemeanor convictions or infractions in Washington State, 2015-2019

|  | **Any violent misdemeanor conviction**  **(N=29086)** | **Infraction**  **(N=1279206)^b^** |
| --- | --- | --- |
| *Age at case file (years)* |  |  |
| Missing | 0 | 2766 |
| Mean (SD) | 34.78 (11.63) | 38.38 (15.10) |
| Range | 18.00 - 95.00 | 18.00 - 99.53 |
| *Sex* |  |  |
| Female | 4901 (16.9%) | 468188 (36.6%) |
| Male | 24131 (83.0%) | 809212 (63.3%) |
| Unknown | 54 (0.2%) | 1806 (0.1%) |
| *Race^a^* |  |  |
| American Indian or Alaskan Native | 744 (2.6%) | 11061 (0.9%) |
| Asian | 775 (2.7%) | 66934 (5.2%) |
| Black | 3605 (12.4%) | 74994 (5.9%) |
| Multiracial | 54 (0.2%) | 3353 (0.3%) |
| Native Hawaiian or Pacific Islander | 11 (0.0%) | 40 (0.0%) |
| Refused | 0 (0.0%) | 31 (0.0%) |
| Unknown | 3321 (11.4%) | 217337 (17.0%) |
| White | 20572 (70.7%) | 905456 (70.8%) |
|  | **Domestic violence-related misdemeanor conviction**  **(N=19487)** | **Infraction**  **(N=1279485)^b^** |
| *Age at case file (years)* |  |  |
| Missing | 0 | 2766 |
| Mean (SD) | 34.82 (11.22) | 38.38 (15.10) |
| Range | 18.00 - 95.00 | 18.00 - 99.53 |
| *Sex* |  |  |
| Female | 2993 (15.4%) | 468229 (36.6%) |
| Male | 16461 (84.5%) | 809450 (63.3%) |
| Unknown | 33 (0.2%) | 1806 (0.1%) |
| *Race^a^* |  |  |
| American Indian or Alaskan Native | 443 (2.3%) | 11066 (0.9%) |
| Asian | 510 (2.6%) | 66936 (5.2%) |
| Black | 2396 (12.3%) | 75012 (5.9%) |
| Multiracial | 28 (0.1%) | 3355 (0.3%) |
| Native Hawaiian or Pacific Islander | 8 (0.0%) | 40 (0.0%) |
| Refused | 0 (0.0%) | 31 (0.0%) |
| Unknown | 2186 (11.2%) | 217378 (17.0%) |
| White | 13913 (71.4%) | 905667 (70.8%) |
|  | **Firearm-related misdemeanor conviction**  **(N=766)** | **Infraction**  **(N=1280070)^b^** |
| *Age at case file (years)* |  |  |
| Missing | 0 | 2766 |
| Mean (SD) | 33.86 (13.62) | 38.38 (15.09) |
| Range | 18.00 - 82.00 | 18.00 - 99.53 |
| *Sex* |  |  |
| Female | 56 (7.3%) | 468318 (36.6%) |
| Male | 707 (92.3%) | 809945 (63.3%) |
| Unknown | 3 (0.4%) | 1807 (0.1%) |
| *Race^a^* |  |  |
| American Indian or Alaskan Native | 24 (3.1%) | 11076 (0.9%) |
| Asian | 30 (3.9%) | 66947 (5.2%) |
| Black | 102 (13.3%) | 75062 (5.9%) |
| Multiracial | 4 (0.5%) | 3356 (0.3%) |
| Native Hawaiian or Pacific Islander | 0 (0.0%) | 40 (0.0%) |
| Refused | 0 (0.0%) | 31 (0.0%) |
| Unknown | 83 (10.8%) | 217444 (17.0%) |
| White | 523 (68.3%) | 906114 (70.8%) |
|  | **Drug/alcohol-related misdemeanor conviction**  **(N=45282)** | **Infraction**  **(N=1278223)^b^** |
| Age at case file (years) |  |  |
| Missing | 0 | 2766 |
| Mean (SD) | 35.01 (13.24) | 38.38 (15.10) |
| Range | 18.00 - 91.00 | 18.00 - 99.53 |
| Sex |  |  |
| Female | 12058 (26.6%) | 467894 (36.6%) |
| Male | 33190 (73.3%) | 808522 (63.3%) |
| Unknown | 34 (0.1%) | 1807 (0.1%) |
| Race^a^ |  |  |
| American Indian or Alaskan Native | 1102 (2.4%) | 11047 (0.9%) |
| Asian | 1160 (2.6%) | 66907 (5.2%) |
| Black | 2602 (5.7%) | 74978 (5.9%) |
| Multiracial | 129 (0.3%) | 3347 (0.3%) |
| Native Hawaiian or Pacific Islander | 6 (0.0%) | 40 (0.0%) |
| Refused | 0 (0.0%) | 31 (0.0%) |
| Unknown | 6714 (14.8%) | 217136 (17.0%) |
| White | 33569 (74.1%) | 904737 (70.8%) |

^a^Ethnicity data were missing for most people and so are not presented

^b^The sample size for the infraction group varied across comparisons because we separately selected each person’s first (“index”) infraction or misdemeanor for each offense/exposure type (thus allowing individuals to contribute observations to each misdemeanor type).

eTable 2. Association between prior misdemeanor conviction and subsequent violent and firearm-related charges and convictions

| *Outcome* | *Count (rate^a^)* | | *sdHR (95% CI)^b^* |
| --- | --- | --- | --- |
|  | **Any violent misdemeanor conviction** | **Infraction** | **Any violent misdemeanor conviction vs. infraction** |
| Subsequent violent charge (any violent crime) | 8198 (126.62) | 5393 (1.35) | 77.95 (74.45, 81.61) |
| Subsequent violent charge (UCR violent crime) | 1568 (20.22) | 1322 (0.33) | 51.31 (43.73, 60.19) |
| Subsequent firearm-related charge | 515 (6.56) | 632 (0.16) | 34.99 (31.08, 39.4) |
| Subsequent violent conviction (any violent crime) | 5355 (77.55) | 1998 (0.5) | 128.27 (118.8, 138.5) |
| Subsequent violent conviction (UCR violent crime) | 197 (2.49) | 189 (0.05) | 43.5 (10.26, 184.39) |
| Subsequent firearm-related conviction | 172 (2.18) | 193 (0.05) | 37.47 (24.77, 56.69) |
|  | **Domestic violence-related misdemeanor conviction** | **Infraction** | **Domestic violence-related misdemeanor conviction vs. infraction** |
| Subsequent violent charge (any violent crime) | 6325 (150.14) | 5438 (1.37) | 92.17 (76.81, 110.59) |
| Subsequent violent charge (UCR violent crime) | 1092 (20.9) | 1331 (0.33) | 52.69 (48.17, 57.64) |
| Subsequent firearm-related charge | 362 (6.83) | 641 (0.16) | 36.11 (28.45, 45.85) |
| Subsequent violent conviction (any violent crime) | 4279 (94.91) | 2020 (0.51) | 155.23 (136.87, 176.07) |
| Subsequent violent conviction (UCR violent crime) | 129 (2.42) | 191 (0.05) | 41.89 (33.09, 53.04) |
| Subsequent firearm-related conviction | 119 (2.24) | 194 (0.05) | 38.27 (29.77, 49.19) |
|  | **Firearm-related misdemeanor conviction** | **Infraction** | **Firearm-related misdemeanor conviction vs. infraction** |
| Subsequent violent charge (any violent crime) | 113 (57.79) | 5570 (1.4) | 35.72 (28.45, 44.86) |
| Subsequent violent charge (UCR violent crime) | 36 (17.2) | 1363 (0.34) | 43.44 (30.58, 61.7) |
| Subsequent firearm-related charge | 47 (22.47) | 646 (0.16) | 121.45 (89.49, 164.81) |
| Subsequent violent conviction (any violent crime) | 51 (24.75) | 2089 (0.52) | 40.81 (30.85, 53.97) |
| Subsequent violent conviction (UCR violent crime) | 7 (3.29) | 195 (0.05) | 57.54 (26.87, 123.24) |
| Subsequent firearm-related conviction | 16 (7.54) | 198 (0.05) | 131.64 (79.18, 218.88) |
| outcome | **Drug/alcohol-related misdemeanor conviction** | **Infraction** | **Drug/alcohol-related misdemeanor conviction vs. infraction** |
| Subsequent violent charge (any violent crime) | 3287 (23.9) | 5488 (1.38) | 16.73 (16.02, 17.48) |
| Subsequent violent charge (UCR violent crime) | 763 (5.36) | 1348 (0.34) | 15.26 (13.89, 16.77) |
| Subsequent firearm-related charge | 354 (2.48) | 644 (0.16) | 15.07 (13.24, 17.16) |
| Subsequent violent conviction (any violent crime) | 1604 (11.43) | 2048 (0.51) | 21.4 (19.41, 23.58) |
| Subsequent violent conviction (UCR violent crime) | 86 (0.6) | 192 (0.05) | 11.73 (9.08, 15.14) |
| Subsequent firearm-related conviction | 138 (0.97) | 192 (0.05) | 19.56 (15.72, 24.34) |

^a^Rate per 1,000 person-years

^b^Estimated from unadjusted competing risk models

eFigure 1. Incidence of subsequent violent and firearm-related charges and convictions among those with prior violent misdemeanor convictions or infractions

misd. = misdemeanor; UCR = uniform crime reporting

eFigure 2. Incidence of subsequent violent and firearm-related charges and convictions among those with prior domestic violence misdemeanor convictions or infractions

DV = domestic violence; misd. = misdemeanor; UCR = uniform crime reporting

eFigure 3. Incidence of subsequent violent and firearm-related charges and convictions among those with prior firearm-related misdemeanor convictions or infractions

misd. = misdemeanor; UCR = uniform crime reporting

eFigure 4. Incidence of subsequent violent and firearm-related charges and convictions among those with prior drug/alcohol-related misdemeanor convictions or infractions

misd. = misdemeanor; UCR = uniform crime reporting
